# BRD3 PROTAC degrader targets H3K18ac to alleviate retinal microglia-driven uveitis

**DOI:** 10.1016/j.isci.2025.114526

**Published:** 2025-12-22

**Authors:** Zhi Zhang, Tianlong Lan, Yongbo Liu, Hui Yang, Nan Shu, Ruonan Li, Wanqian Li, Qian Zhou, Peizeng Yang, Yu Rao, Shengping Hou

**Affiliations:** 1 Chongqing Key Laboratory of Ophthalmology, Chongqing Eye Institute, Chongqing Branch of National Clinical Research Center for Ocular Diseases, The First Affiliated Hospital of Chongqing Medical University, Chongqing 400016, China; 2 Beijing Institute of Ophthalmology, Beijing Tongren Eye Center, Beijing Ophthalmology & Visual Sciences Key Laboratory, Beijing Tongren Hospital, Capital Medical University, Beijing 100730, China; 3 MOE Key Laboratory of Protein Sciences, School of Pharmaceutical Sciences, MOE Key Laboratory of Bioorganic Phosphorus Chemistry and Chemical Biology, Tsinghua University, Beijing 100084, China; 4 Chongqing Medical and Pharmaceutical College, Chongqing 401331, China

**Keywords:** Ophthalmology, Biological sciences, Epigenetics

## Abstract

Uveitis is a sight-threatening intraocular inflammation in which the proinflammatory immune response driven by retinal microglia is a key contributor. Proteolysis targeting chimera (PROTAC) targeting bromodomain and extraterminal (BET) proteins has shown therapeutic effects in certain inflammatory diseases or tumors, but their effects on uveitis remain elusive. Our research demonstrated that PROTAC D072 reduced intraocular inflammation *in vivo* and inhibited proinflammatory microglia in both uveitis retina and lipopolysaccharide (LPS) treated mouse microglia cell line BV2. Drug target verification revealed that D072 specifically degraded BRD3 but did not significantly affect BRD2 or BRD4. Mechanistically, BRD3 degradation resulted in reduced H3K18ac, and CUT&Tag analysis revealed changes in the occupancy of several proinflammatory and metabolism-related genes. Furthermore, histone deacetylases (HDACs) partially regulate the H3K18ac level following BRD3 degradation. Overall, we identified D072 as a specific degrader of BRD3 in the murine system that can inhibit proinflammatory microglia in autoimmune uveitis, potentially providing a therapeutic approach for uveitis.

## Highlights

• A PROTAC named D072 selectively degrades BRD3 in mice
• BRD3 is involved in the proinflammatory process of retinal microglia in EAU
• BRD3-H3K18ac modulated chromatin state of inflammation and metabolism gene
• HDAC-driven H3K18ac change controlled by BRD3 to inhibit CCL5 signaling

## Graphical abstract

**Figure:**
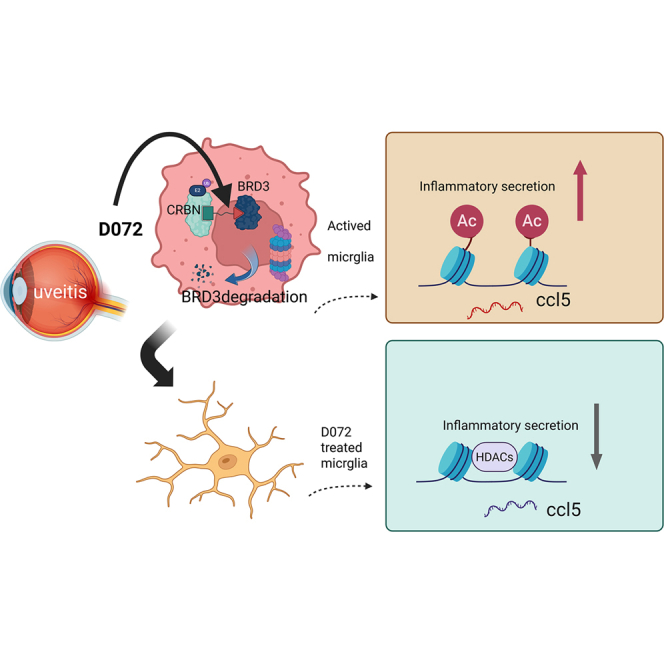

## Introduction

Uveitis is one of the most common intraocular inflammatory diseases and is a significant cause of visual impairment and blindness. It is generally classified as infectious uveitis caused by pathogens such as bacteria, viruses, and parasites or non-infectious uveitis that is associated with autoimmune diseases, including Behcet’s disease, sarcoidosis, and Vogt-Koyanagi-Harada disease.^1^^,^^2^^,^^3^ Current therapeutic approaches for uveitis, such as corticosteroids and immunosuppressive agents, are effective in reducing inflammation but are associated with significant side effects, particularly with long-term use.^3^ These treatments broadly suppress the immune system rather than targeting the underlying causes. This underscores the necessity to develop novel therapeutics with high efficacy and safety.

The bromodomain and extraterminal (BET) family of proteins controls the transcription of a wide range of proinflammatory and immunoregulatory genes by recognizing acetylated histones and recruiting transcription factors.^4^ BET proteins consist of testis-specific isoform BRDT and three abundantly and ubiquitously expressed BRD2, 3, and 4 proteins. The dysfunction of BET proteins is involved in many physiological and pathological processes and has become an important therapeutic target for immune and inflammatory diseases.^5^ Over the past decades, much research has revealed that inhibition of BET proteins can reduce inflammation in rheumatoid arthritis^6^ and neuroinflammation associated CNS (central nervous system) diseases.^7^ Proteolysis-targeting chimera (PROTAC) is a useful tool that specifically degrades unwanted proteins in cells, it does not require a deep hydrophobic binding pocket or active site and thus offers significant advantages over conventional small-molecule inhibitors. BET-targeting PROTACs have shown potential therapeutic effects in inflammation-mediated diseases, including stroke, Alzheimer’s disease, and age-related macular degeneration (AMD).^8^ However, the BET degradation of PROTACs used in most studies lacks specificity, often involving dual degradation or pan-BET degradation, which may lead to severe consequences.^9^^,^^10^ For example, it has been reported that BRD2 and BRD4 knockout mice can cause embryonic lethality.^11^ Other studies have shown that BRD4 degradation PROTACs affect the hematopoietic function of adult mice and reduce the number of intestinal stem cells, showing the potential side effects of BRD4 degraders.^12^^,^^13^ These results emphasized the importance of a comprehensive evaluation of downstream effects after BET proteins degradation. Despite evidence indicating the beneficial role of BET degradation in regulating autoimmune response, its function in autoimmune uveitis remains unclear.

Experimental autoimmune uveitis (EAU) is an animal disease model established to better characterize human autoimmune uveitis. It is widely used to delineate the pathophysiological processes of ocular autoimmunity and develop therapeutic approaches.^14^ In addition to peripheral immune cells, increasing evidence shows that microglia are involved in the occurrence and development of autoimmune uveitis. Microglia displayed a typical ameboid-like activation during the peak of EAU disease. Activated microglia can harm the affected tissue due to their altered functional states, including phagocytosis, antigen presentation, and production of inflammatory factors. Controlling microglial function by immunomodulatory therapy under disease conditions is a major focus of microglia research.^15^^,^^16^

In this study, we synthesized a PROTAC library and screened a specific BRD3 degrader, PROTAC D072, which can alleviate the clinical and pathological severity of EAU and decrease the activation levels of retinal microglia. Western blot and RT-qPCR results showed that the protein and mRNA levels of some proinflammatory genes were decreased after D072 treatment in LPS-stimulated BV2 cells and mouse retina. Mechanistically, we found that D072-induced BRD3 degradation resulted in changes in histone acetylation modification, especially Histone H3 acetyl Lys 18 (H3K18ac), which was significantly downregulated after D072 treatment. Cleavage Under Targets and Tagmentation (CUT&Tag) data further revealed that H3K18ac occupied the promoter regions of metabolism-related genes and proinflammatory genes, and D072 treatment suppressed their mRNA expression. Finally, we found that histone deacetylases (HDACs) partially regulated the H3K18ac level after BRD3 degradation, and *Ccl5* may be its direct target. In conclusion, a specific BRD3 degrader PROTAC D072 was identified, which showed protective effects for uveitis by inhibiting the BRD3/H3K18ac/CCL5 axis in proinflammatory retinal microglia. This study validates BRD3 protein degradation as a promising clinical strategy against eye inflammation and demonstrates the feasibility of treating autoimmune uveitis with PROTACs.

## Results

### D072 shows an anti-inflammatory effect in BV2 cells

**Figure 1:**
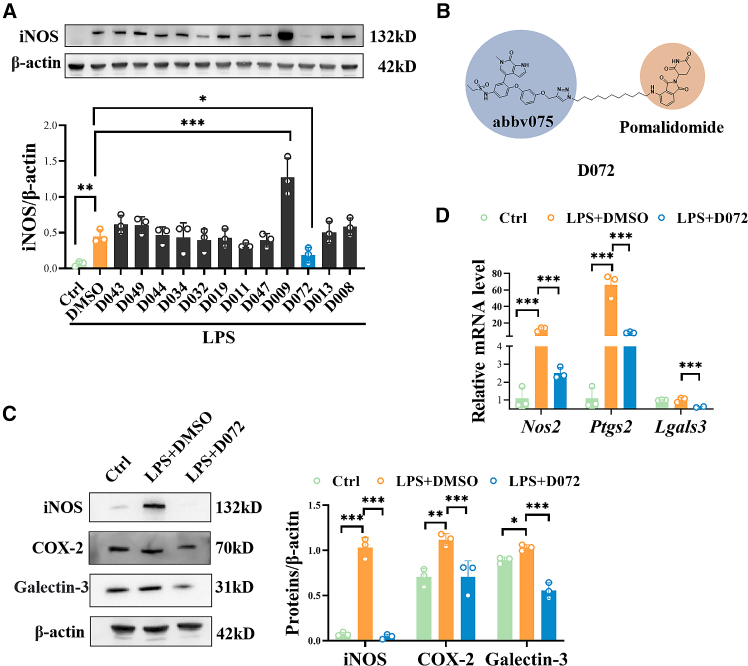
D072 has a potential anti-inflammatory effect in microglia (A) The inhibition results of INOS protein after LPS stimulation of BV2 cells by various drugs. (B) The chemical structure formula of D072. (C and D) D072 can inhibit the expression of INOS, COX-2, and Galectin-3 proteins and their mRNA levels in BV2 cells after LPS stimulation. (A) Quantification of the protein levels of iNOS of 12 PROTACs. Western blotting was performed to evaluate the inhibition of iNOS expression of LPS-stimulated BV2 cells after 12 compounds treatment for 6 h. (*n* = 3/group; mean ± SD; *∗p* < 0.05, *∗∗p* < 0.01, *∗∗∗p* < 0.001; one-way ANOVA). (B) Schematic diagram of the chemical structure of D072. (C) Left: representative western blot images of iNOS, COX2, and Galectin-3 after D072 treatment in LPS-stimulated BV2 cells. Right: quantification of the protein levels of iNOS, COX2, and Galectin-3 after D072 treatment LPS-stimulated BV2 cells. (*n* = 3/group; mean ± SD; *∗p* < 0.05, *∗∗p* < 0.01, *∗∗∗p* < 0.001; one-way ANOVA). (D) Quantification of the mRNA levels of iNOS, COX2, and Galectin-3 after D072 treatment for 6 h in LPS-stimulated BV2 cells (*n* = 3/group; mean ± SD; *∗∗p* < 0.01; one-way ANOVA).

Our previous works have demonstrated the critical role of microglia in autoimmune uveitis.^17^^,^^18^ To identify potential anti-inflammatory molecules targeting microglia, we screened compounds from a synthesized PROTAC library using LPS-stimulated murine microglial BV2 cells. First, the anti-inflammatory effects of 12 compounds were evaluated by analyzing their effect on the expression of iNOS. iNOS is a classical, widely used proinflammatory indicator, and its expression is upregulated in EAU retina and microglia under inflammatory conditions.^15^^,^^16^^,^^17^^,^^18^ As shown in Figure 1A, western blot analysis revealed that D072 (Figures 1B and S1) had the strongest inhibitory effect on iNOS protein expression. Additionally, RT-qPCR results showed that D072 treatment significantly reduced the mRNA level of iNOS in LPS-treated BV2 cells (Figure S2A), suggesting that D072 may inhibit iNOS expression by reducing its mRNA level. In the cell counting kit 8 (CCK 8) assay, we observed relatively high cell viability of human microglia cell line HMC3 under a very high concentration treatment of D072 (10 μM; Figure S2F).

Subsequently, we further explored the inhibitory mechanism of D072 on microglial inflammatory activation. In the beginning, the CCK 8 assay was performed to select an appropriate D072 concentration to exclude its effect on cell viability. As shown in Figure S2B, 10 nM D072 showed no statistically significant effect on cell activity and was selected for subsequent experiments. BV2 cells were treated with LPS to simulate inflammatory activation, followed by the addition of 10 nM D072 or an equal volume of solvent as the vehicle group. The protein levels and corresponding mRNA levels of *Nos2*, *Ptgs2*, and *Lagals3* were measured by western blot and RT-qPCR, respectively. It is shown in Figure 1C that the proteins of iNOS, COX-2, and Galetcin-3, which were increased by LPS stimulation in the vehicle group, were significantly inhibited in the D072 treatment group. Although *Lagals3*, the Galetcin-3 mRNA level did not change significantly between the control group and the LPS+DMSO group, the D072 treatment reduced the mRNA expression levels of all indicators (Figure 1D). These findings suggest that D072 may exert a therapeutic effect by regulating the activation of proinflammatory microglia.

### D072 inhibited the activation of retinal microglia and alleviated the retinal severity of EAU

**Figure 2:**
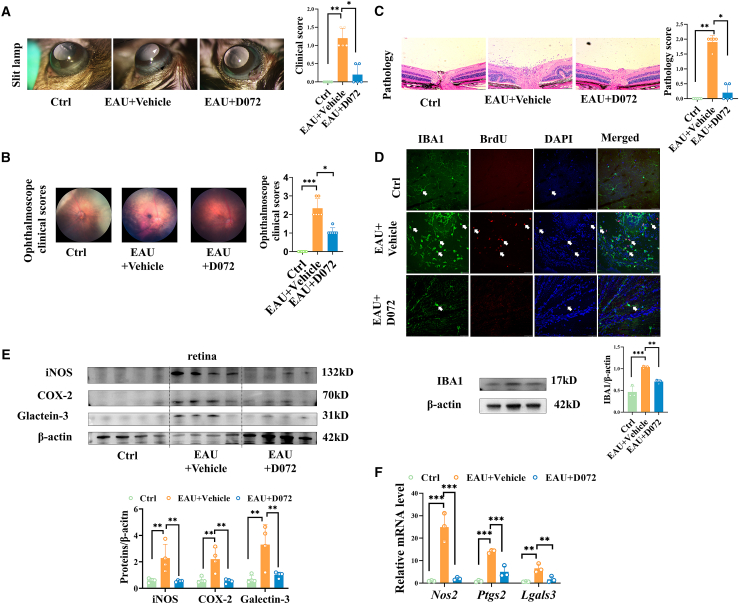
D072 alleviates the retinal severity of EAU and activation of retinal microglia (A–C) On the 9^th^ day of EAU induction, a intravitreal injection was performed using D072 (1 μL, 300 μM). Samples were collected on the 14^th^ day, and evaluations were conducted for the anterior segment, fundus, and pathological scoring was performed using H&E staining. (D) Retinal immunofluorescence staining with IBA1 (green), DAPI (blue), and BrdU (red) was used to detect the proliferation level of retinal microglia. Determination of IBA1 protein levels in retinal tissue. (E) The expression levels of retinal inflammatory factors INOS, COX-2, and Galectin-3 protein and mRNA in the normal group, the EAU group, and the D072 treatment group. (A) Representative pictures of slit lamp photography at day 14 of each group, with quantification of clinical scores (*n* = 5/group; median ± interquartile; *∗p* < 0.05, *∗∗p* < 0.01; Mann-Whitney U test). (B) Representative fundus pictures of posterior pole of each group on day 14 (*n* = 6/group; median ± interquartile; ∗*p* < 0.05, ∗∗*p* < 0.01; Kruskal-Wallis). (C) Representative H&E staining sections of the retina at day 14 of each group (scale bars, 50 μm), with quantification of pathological scores (*n* = 5/group; median ± interquartile; ∗*p* < 0.05, ∗∗*p* < 0.01; Kruskal-Wallis). (D) Upper: representative immunostaining images of microglia (IBA1, green), (DAPI, blue), and (BrdU, red) in retinal flat mounts (*n* = 3; scale bars, 100 μm). Lower: representative images of western blotting results for microglial marker IBA1 in whole retinal extracts and corresponding statistics (*n* = 3/group; mean ± SD; *∗∗p* < 0.01, *∗∗∗p* < 0.001; one-way ANOVA). (E) Representative images of western blotting results for inflammatory factors iNOS, COX-2, and Galectin-3 in whole retinal extracts. (F) Quantification of the protein levels of iNOS, COX-2, and Galectin-3 (*n* = 4/group; mean ± SD; *∗∗p* < 0.01; one-way ANOVA) and the corresponding mRNA levels (*n* = 3/group; mean ± SD; *∗∗p* < 0.01, ∗∗∗*p* < 0.001; one-way ANOVA) in the retinas of these mice.

Next, the *in vivo* effect of D072 was investigated through its intravitreal injection in the EAU mouse model. The anterior segments of the eyes of mice were evaluated by a slit-lamp on day 9 after EAU immunization. Mice with similar ciliary hyperemia and/or anterior chamber inflammation were randomly assigned to the D072 intervention group (1 μL, 300 μM) or the vehicle group, and the rest were excluded. Previous studies have shown that within 7–10 days after EAU induction, microglia begin to polarize and contact with peripheral leukocytes.^19^ Therefore, we speculate that although D072 may have an impact on peripheral immune cells, its use on the 9^th^ day might mainly affect the function of microglia. On the 14^th^ day after immunization, clinical scoring was performed based on the anterior segment, the ophthalmoscope clinical scoring was performed based on fundus images, and the pathological scoring was conducted by H&E-stained histopathological sections. Compared with the vehicle group, the anterior inflammation of EAU mice in the D072-treated intervention group was less severe, and the clinical score was significantly reduced (Figure 2A). The posterior pole focal lesions, tortuous blood vessels, and the ophthalmoscope clinical scores were all improved (Figure 2B). H&E-stained histopathological sections also confirmed that there were fewer retinal folds or inflammatory cell infiltrations in the retinas of EAU mice treated with D072 (Figure 2C).

In addition, the cell morphology was identified by retinal immunofluorescence staining of the microglial cell marker IBA1. We found that the microglial cells in the vehicle group showed an increased proliferation, with most cells exhibiting an amoeboid morphology characteristic of activated microglia. In contrast, most of the EAU retinal microglia in the D072 treatment group showed branched morphology, similar to the steady-state microglia in the control group (Figure 2D). Further BrdU staining indicated that the D072 treatment inhibited the proliferation and activation of microglia in EAU (Figure 2D). Western blot analysis of retinal protein also showed that the expression of IBA1 in the D072 treated group was lower than that in the EAU vehicle group, indicating that the proliferation and activation of microglia were inhibited (Figure 2D). These clinical, histopathological, and IBA1 staining evaluations indicated that D072 treatment significantly reduced the retinal tissue damage and microglial activation in EAU mice.

On the 14^th^ day after immunization, the mouse retinas were collected for RT-qPCR and western blot analysis to verify whether D072 could regulate the level of retinal inflammation. The results in Figures 2E and 2F showed that iNOS, COX-2, and Galecitn-3 mRNA and protein levels in the retinas of mice treated with 300 μM D072 were significantly lower than those of mice in the vehicle group, which are consistent with our *in vitro* results. The above findings further illustrated that D072 could alleviate the inflammatory factors of EAU and inhibit microglia activation *in vivo*.

To detect the potential side effects of D072, we measured the level of retinal cleaved caspase3. The results showed that the cleaved caspase3 in the D072 treatment group was comparable to that in the control group, suggesting that D072 has few side effects on the retina (Figure S4B).

### D072 exerts anti-inflammatory effects by specifically degrading BRD3

**Figure 3:**
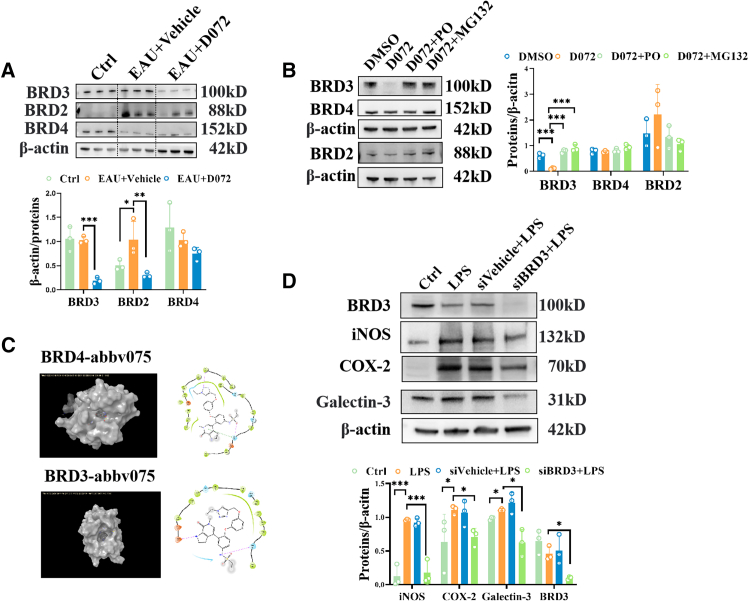
D072 exerts anti-inflammatory effects by specifically degrading BRD3 (A) The expressions of BRD2, BRD3, and BRD4 in control group, EAU group, and D072 treatment group of the retina. (B) The *in vitro* expression of BRD2, BRD3, and BRD4 under the intervention of the ubiquitin pathway. (C) Molecular docking of BRD3 and BRD4. (D) After knockdown of BRD3 in BV2 cells, the protein expression levels of INOS, COX2, and Galectin-3 receptor in response to LPS stimulation were measured. (A) Upper: representative western blot images of retinal BRD2, BRD3, and BRD4 in Control group, EAU+Vehicle group, and EAU+D072 group, respectively. Lower: quantification of the relative fold changes of the left (*n* = 3/group; mean ± SD; *∗p* < 0.05, *∗∗p* < 0.01, *∗∗∗p* < 0.001; one-way ANOVA). (B) Left: representative western blot images of BRD2, BRD3, and BRD4 in each group in BV2. Right: quantification of the relative changes of the left (*n* = 3/group; mean ± SD; *∗∗∗p* < 0.001; one-way ANOVA). (C) The binding modes of BRD3 and BRD4 with D072. (D) Western blotting analysis of the protein levels of iNOS, COX-2, Galectin-3, and BRD3 in different groups. Left: representative western blot images of iNOS, COX-2, Galectin-3, and BRD3 in different groups. Right: quantification of the relative fold changes of the left (*n* = 3/group; mean ± SD; *∗p* < 0.05, *∗∗p* < 0.01, *∗∗∗p* < 0.001; one-way ANOVA).

Then, we further investigated the functional mechanisms of D072. As shown in Figure 1B, D072 is a BET PROTAC composed of a CRBN E3 ubiquitin ligase binding moiety pomalidomide and a BET inhibitor abbv075. This suggests that D072 may exert its anti-inflammatory effect by degrading BET protein. The BET family proteins, consisting of BRD2, BRD3, BRD4, and BRDT, are widely acknowledged as epigenetic readers and transcriptional regulators. Since BRDT is testis-specific, it was excluded from this study. To identify the target of D072, we first detected the expression level of BRD2, BRD3, and BRD4 proteins in previous EAU retinal samples. It is shown in Figure 3A that BRD3 protein of EAU group was significantly decreased after D072 treatment, while no significant change was observed in BRD3 expression between the control group and EAU vehicle group. BRD2 expression was upregulated in the EAU vehicle group and was downregulated after D072 treatment, while BRD4 showed no statistically significant changes in all three groups. It is noteworthy that these results were obtained 5 days after the intravitreal injection of D072 into the retina. Drug-induced protein degradation may occur in a very short time.^20^ Therefore, we use BV2 to verify our findings. We introduced pomalidomide to compete with D072 for the binding of E3 ubiquitin ligase CRBN and used MG132 to interfere with the proteasome pathway to confirm the degradation mechanism. BV2 cells treated with D072 at 1 μM for 4 h showed a significant reduction in BRD3 protein levels but had no effect on BRD2 and BRD4. As expected, BRD3 degradation was prevented by pomalidomide and MG132, respectively (Figure 3B). These results suggest that D072 mediates the specific degradation of BRD3 through CRBN, and the changes in BRD2 in the retina may be a subsequent effect of BRD3 rather than a direct degradation by D072 interaction.

To further understand the selectivity of D072 for BRD3, the binding modes of BRD3 and BRD4 with D072 were analyzed by molecular docking. Molecular docking analysis of the ABBV075 moiety of D072 with BRD4 revealed that its hexacyclic fused five-membered ring structure engages in π-π stacking interactions with His438, while the sulfonamide group forms critical hydrogen bonds with the same residue. These dual interactions rigidly anchor the compound’s binding conformation, positioning the triazole ring deep within the BRD4 binding pocket. Subsequent derivatization of the triazole ring with linker-E3 ligase ligand conjugation would disrupt this stable binding configuration, thereby hindering the formation of a functional ternary complex (D072-BRD4-CRBN) required for proteasomal degradation.

In contrast, BRD3 lacks the equivalent histidine residue (His438 in BRD4). Molecular modeling demonstrated that the triazole ring of D072 occupies a solvent-exposed region in BRD3, allowing unimpeded linker extension for CRBN recruitment. This structural distinction enables D072 to form a stable ternary complex with BRD3 and CRBN, resulting in selective degradation of BRD3 protein. This differential binding mechanism between BRD4 and BRD3 provides a structural basis for the observed isoform-selective degradation profile (Figure 3C).

Furthermore, to determine the regulatory function of BRD3 on inflammation, we treated BV2 cells with the BRD3-targeting siRNAs or BRD3 overexpression lentivirus and assessed the protein levels of iNOS, COX-2, and Galectin-3. Knockdown of BRD3 by the selected siRNA (Figure S2C) led to the reduction of these proinflammatory markers, while BRD3 overexpression reversed this effect (Figures S2D and S2E), further indicating that the anti-inflammatory effect of D072 may be mainly mediated by degrading BRD3 (Figure 3D).

### The degradation of BRD3 changed the modification of histone acetylation

**Figure 4:**
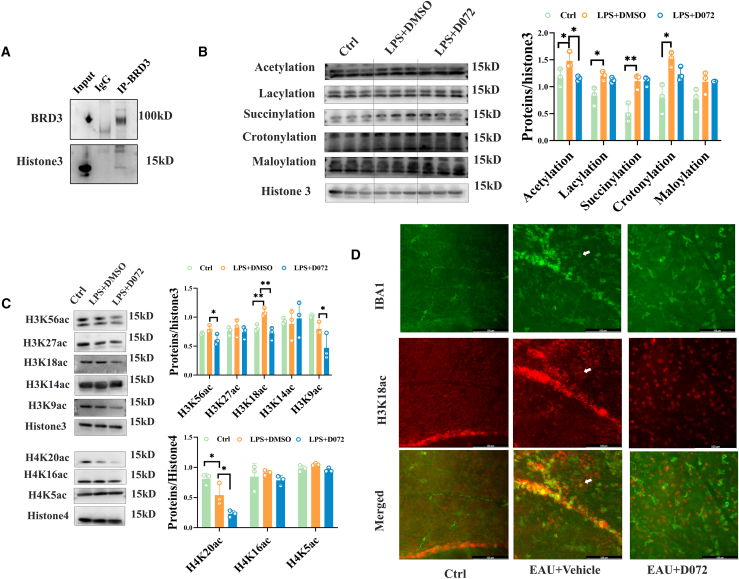
The degradation of BRD3 changed the modification of histone acetylation (A) The CO-1P experiment of BRD3 and histone 3. (B) Changes in various histone modifications after D072 treatment. (C) The alteration of acetylation modification under drug treatment. (D) The changes in H3K18ac expression of retinal microglia under drug treatment. (A) Representative western blot images of immunoprecipitation IgG and BRD3 and IB: Histone3. (B) Left: representative western blot images of pan-antibody of different modifications, including acetylation, lactylation, succinylation, crotonylation, malonylation, and Histone 3. Right: quantification of the relative changes of the left (*n* = 3/group; mean ± SD; *∗p* < 0.05, *∗∗p* < 0.01; one-way ANOVA). (C) Left: representative western blot images of different site-specific acetylated antibodies. Right: quantification of the relative fold changes of the left (*n* = 3/group; mean ± SD; *∗p* < 0.05, *∗∗p* < 0.01; one-way ANOVA). (D) Representative immunofluorescent staining images of microglia (IBA-1, green) and H3K18ac (red) in the retinas of control, EAU+Vehicle, and EAU+D072 mice (white arrows represent double-positive cells). (Scale bars, 100 μm).

Next, we investigated the mechanism of anti-inflammatory effect under BRD3 degradation. BRD3 is a member of the BET family, which primarily recognizes acetylation sites through BD1 and BD2 domains, so it is considered to be the recognition protein for histone acetylation modification.^21^ We first detected the binding of BRD3 and histone 3 by co-immunoprecipitation experiment and found that BRD3 had potential binding ability with histone 3 (Figure 4A). Based on this, we speculated that BRD3 degradation might expose previously bound acetylation sites, leading to subsequent modifications mediated by acetylases and deacetylases. To test this hypothesis, we employed a pan-antibody system to detect changes in different histone modifications. As shown in Figure 4B that acetylation, succinylation, lactation, and crotonylation of histones were all upregulated under LPS treatment, while only acetylation was decreased following D072 treatment, with the other modifications remaining largely unchanged (Figures 4B and S3A). Further screening of histone acetylation sites revealed that multiple acetylation modification sites changed under D072 treatment, including H3K56ac, H3K18ac, H3K9ac, H4K20ac, and H4K8ac, but only H3K18ac was upregulated under LPS stimulation (Figures 4C and S3B). Similarly, through fluorescent co-staining of IBA1 and H3K18ac in the retina, it was found that H3K18ac was significantly upregulated in amoeba microglia in the EAU vehicle group, and the expression level of H3K18ac was reduced after D072 treatment (Figure 4D). Our *in vitro* and *in vivo* results suggested that H3K18ac site was associated with microglial activation and that D072 treatment could reduce the expression of H3K18ac, suggesting that D072 may exert its anti-inflammatory effects by modulating downstream target genes of H3K18ac.

### H3K18ac regulates genes associated with inflammation and metabolism

**Figure 5:**
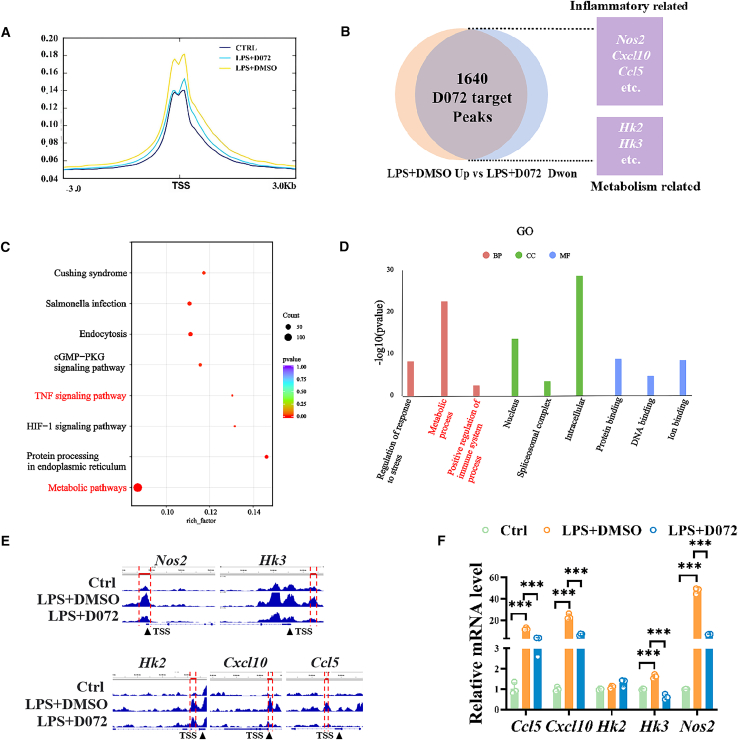
H3K18ac regulates genes associated with inflammation and metabolism (A) The peak positions of transcription initiation sites in Control group, LPS group, and D072 treatment group experiments *in vitro*. (B) Venn diagram screening for potential regulatory genes of D072. (C and D) KEGG and GO analysis of the D072 regulatory gene. (A) The peak on TSS (transcription start site) in Control, LPS+DMSO, and LPS+D072 groups in BV2. (B) A Venn diagram showed the shared targets between up-regulated peak in LPS+DMSO group and downregulated in LPS+D072 group. (C and D) KEGG and GO analyses of the 1460 targets. (E) The peaks of *Nos2*, *Hk3*, *Hk2*, *Ccl5*, and *Cxcl10* under different treatments were shown. (F) Quantitative statistics of mRNA level of iNOS, HK2, HK3, CCL5, and CXCL10. (*n* = 3/group; mean ± SD; *∗∗∗p* < 0.001; one-way ANOVA).

H3K18ac is an epigenetic modification that causes chromatin opening, thereby promoting transcription. Previous studies have reported that H3K18ac is related to the expression of endodermal differentiation genes,^21^^,^^22^ but the role of H3K18ac in the proinflammatory process of microglia remains unclear. To further analyze the downstream target genes regulated by H3K18ac, we performed H3K18ac CUT&Tag to identify candidate genes modulated by H3K18ac across different treatment conditions, including the control group, DMSO+LPS group, and D072+LPS group. In Figure 5A, our initial results revealed that, compared to the control group, the LPS+DMSO group presented a higher H3K18ac binding peak at the transcription initiation site. The peak was reduced to a level comparable to the control group after D072 treatment, which was consistent with the trend of H3K18ac protein expression detected by western blot.

To further identify the potential regulatory genes influenced by D072, we screened genes related to peaks in promoter binding regions. In Figure 5B, we identified 3,843 genes with upregulated peaks in the LPS+DMSO group compared to the control group and 2,162 genes with downregulated peaks in the D072+LPS group compared to the LPS+DMSO group. Importantly, 1,460 of these genes showed shared patterns of being upregulated in the LPS+DMSO group and downregulated after D072 treatment, indicating that they are potential target genes for the functioning of PROTAC D072.

Subsequently, we conducted KEGG pathway analysis on the 1460 identified genes and found that these pathways included metabolism, HIF-1 signaling, TNF-α signaling, cGMP-pPKG signaling, Endocytosis, Salmonella infection, Cushing’s syndrome, and protein processing pathways in the endoplasmic reticulum. Gene Ontology (GO) analysis further identified significant enrichment of genes related to metabolism and positive regulation of immune system processes (Figures 5C and 5D). Given previous reports that HK2 and HK3 are primarily expressed in microglia, we selected *Hk2*, *Hk3*, *Nos2*, *Cxcl10*, and *Ccl5* as representative genes for further investigation, focusing on their roles in metabolism and inflammation. The H3K18ac binding peaks of these genes at the transcription initiation sites were significantly enhanced in LPS+DMSO group but decreased after D072 treatment (Figure 5E). This indicated that the upregulation of H3K18ac in the LPS+DMSO group increased the chromatin accessibility of these genes, which may have promoted their transcription, while D072 treatment reduced the accessibility of these genes, thereby inhibiting the transcription of these genes.

Chromatin openness is an important aspect affecting transcription, but it also requires the binding of corresponding transcription factors to promote transcription. RT-qPCR was further used to detect the mRNA levels of these target genes. It is shown in Figure 5F that the mRNA levels of *Hk3*, *Nos2*, *Ccl5*, and *Cxcl10* were increased in the LPS+DMSO group, which aligned with the observed H3K18ac enrichment peaks. This indicates that their gene expression may be affected by the epigenetic modification of H3K18ac. In contrast, *Hk2* expression showed no significant changes, which may be attributed to the lack of the corresponding transcription factors required for its transcriptional activation.

### The H3K18ac target gene is partially regulated by HDACs

**Figure 6:**
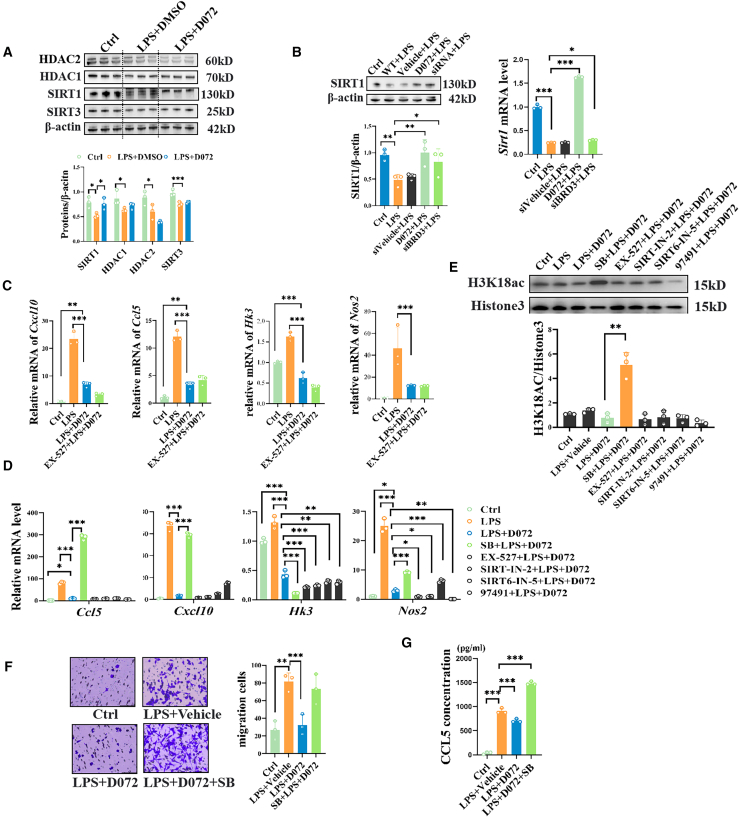
The regulation of H3K18ac by BRD3 degradation is mediated by HDACs, and CCL5 is its target (A) The expression of several deacetylase enzymes (HDAC1, HDAC2, Sirt1, and Sirt3) in control group, LPS group, and D072 treatment group experiments *in vitro*. (B) The expression levels of SIRT1 under various treatments (LPS stimulation, D072 treatment, or siBRD3 knockdown), Sirt1 changes in proteins and mRNA. (C) The mRNA levels of cxcl10, ccl5, HK3, and nos2 in control group, LPS group, D072 treatment group, D072 + SIRT1 inhibitor (EX-527 treated) group *in vitro*. (D) The expression levels of CCL5, CXCL10, HK3, and NO2 under the intervention of each group of HDAC inhibitors. (Inhibitor terminology and function: SB: sodium butanoate [HDACs inhibitor], EX-527 [SIRT1 inhibitor], SIRT-IN-2 [SIRT1, 2, 3 inhibitors], SIRT6-IN-5 [SIRT6 inhibitor], and 97491 [SIRT7 inhibitor]). (E) The expression changes of H3K18ac under the intervention of each group of HDAC inhibitors. (Inhibitor terminology and function: SB: sodium butanoate [HDACs inhibitor], EX-527 [SIRT1 inhibitor], SIRT-IN-2 [SIRT1, 2, 3 inhibitors], SIRT6-IN-5 [SIRT6 inhibitor], and 97491 [SIRT7 inhibitor]). (A) Upper: representative western blot images of HDAC1, HDAC2, SIRT1, and SIRT3 in each group. Lower: quantification of the relative changes of the left (*n* = 3/group; mean ± SD; ns, *p* > 0.05, *∗p* < 0.05, *∗∗∗p* < 0.001; one-way ANOVA). (B) Left: representative western blot images and the corresponding quantification of the relative protein levels of SIRT1 in each group. Right: quantification of the relative mRNA changes of the SIRT1 in different groups. (*n* = 3/group; mean ± SD; *∗p* < 0.05, *∗∗p* < 0.01, *∗∗∗p* < 0.001; one-way ANOVA). (C) Quantification of the mRNA level of *Cxcl10*, *Ccl5*, *Hk3*, and *inos* after SIRT1 inhibitor EX-527 treatment. (*n* = 3/group; mean ± SD; *∗∗p* < 0.01, *∗∗∗p* < 0.001; one-way ANOVA). (D) Quantification of the mRNA level of *Cxcl10*, *Ccl5*, *Hk3*, and *inos* under different HDAC inhibitor treatment. (*n* = 3/group; mean ± SD; *∗p* < 0.05, *∗∗p* < 0.01, *∗∗∗p* < 0.001; one-way ANOVA). Inhibitor terminology and function: SB: sodium butanoate (HDACs inhibitor), EX-527 (SIRT1 inhibitor), SIRT-IN-2 (SIRT1, 2, 3 inhibitors), SIRT6-IN-5 (SIRT6 inhibitor), and 97491 (SIRT7 inhibitor). (E) Quantification of the relative H3K18ac protein level under each treatment. (*n* = 3/group; mean ± SD; *∗∗∗p* < 0.001; one-way ANOVA). (F) Left: transwell images of different groups; scale bars: 100 μm. Right: quantification of migration cells under each treatment. (*n* = 3/group; mean ± SD; *∗∗∗p* < 0.001; one-way ANOVA). (G) Quantification statistics concentration of CCL5 in each group. (*n* = 3/group; mean ± SD; *∗∗∗p* < 0.001; one-way ANOVA).

Finally, we investigated the role of deacetylases in regulating H3K18ac following BRD3 degradation. We examined the expression levels of a range of deacetylases and found that SIRT1 was upregulated after D072 treatment, while other proteins, including HDAC1, HDAC2, SIRT1, and SIRT3, did not exhibit significant changes in expression (Figure 6A). The same result was obtained by siRNA knockdown of BRD3, and RT-qPCR showed that the upregulation of SIRT1 was achieved by upregulating its mRNA. Similar to our results, BET inhibitor JQ1 can also induce SIRT1 in various cell types.^23^ Therefore, we speculated that upregulated SIRT1 may be responsible for decreased H3K18ac levels (Figure 6B). We then treated the cells with a specific SIRT1 inhibitor EX-527 interestingly, contrary to previous reports, our results showed that inhibiting SIRT1 did not lead to obvious upregulation of inflammatory genes.^24^^,^^25^ These findings suggest that, at least in our experimental system, the elevated SIRT1 does not regulate inflammation at the transcriptional level and is unlikely to directly affect H3K18ac as an enzyme (Figure 6C).

To further investigate the role of deacetylases in regulating H3K18ac, we employed a more direct approach by using inhibitors to interfere with deacetylase function. It was found that the HDACs inhibitor sodium butyrate treatment group could upregulate the mRNA levels of CCL5, CXCL10, and iNOS inhibited by D072 treatment and had no significant induction effect on HK3 (Figure 6D). Meanwhile, Figure 6E showed the western blot results that sodium butyrate also strongly enhanced H3K18ac modification. Given that the CCL5 mRNA was most prominently upregulated under sodium butyrate treatment and has been previously implicated in promoting proinflammatory and pro-migration effects in microglia,^26^ we therefore conducted the further investigation. ELISA analysis was performed to detect CCL5 protein levels in the BV2 cell culture medium, and it was found that CCL5 expression was significantly elevated in the LPS+DMSO group compared to the control group, while CCL5 levels were reduced in the LPS+D072 group compared to the LPS+DMSO group. Sodium butyrate treatment restored CCL5 levels in the LPS+D072 group, suggesting that HDACs may play a role in regulating H3K18ac levels and consequently affect CCL5 expression. Further migration assays confirmed that D072 treatment inhibited the migration of LPS-stimulated BV2 cells, and this effect was reversed by sodium butyrate (Figures 6F and 6G). These findings demonstrated that the BRD3-H3K18ac axis plays a crucial role in the inflammatory response of microglia by regulating CCL5 expression, with HDACs involved in the modulation of this pathway. Therefore, D072’s therapeutic effects in uveitis may be mediated through the BRD3-H3K18ac axis and its impact on inflammatory microglia.

## Discussion

Persistent inflammation and active proinflammatory microglia have been identified as the main pathogenic factor in a variety of retinal diseases,^27^ including uveitis. It has been reported that drugs that inhibit inflammation or reduce retinal microglia activity showed therapeutic potential in EAU. This study first provides *in vitro* and *in vivo* evidence for the anti-inflammatory effects of BET-targeting PROTAC D072. Specifically, intravitreal administration of D072 improves clinical and histopathological outcomes in EAU mice and alleviates inflammation of BV2 cells under LPS treatment *in vitro*. At present, PROTACs targeting the BET family, such as QCA570 and dBET6,^28^^,^^29^ mainly target BRD4. For example, dBET6 has been reported to play a therapeutic role in age-related macular degeneration by inhibiting the cGAS-STING pathway.^30^ However, these PROTACs typically utilize JQ1 as a warhead, leading to off-target effects that result in the degradation of other BET family members like BRD2 and BRD3. In contrast, BRD3 is a relatively underexplored member of the BET family.

In our work, we developed a novel PROTAC D072 that exerts its anti-inflammatory effect in the murine system by only degrading BRD3. Taking this advantage, we could study the effect of BRD3 itself and avoid the ambiguous results caused by BRD2 or BRD4 changes. In our single-cell data analysis, although the mRNA level of BRD3 was slightly upregulated in the EAU retina (Figure S4A), there was no statistical difference in the protein expression of BRD3 in EAU retina and LPS-stimulated BV2 cells. Due to the low number of retinal microglial cells obtained through flow sorting, as well as the lack of suitable commercial BRD3 fluorescent dyes, we were unable to further detect the BRD3 protein level in retinal microglial. In the future, by constructing gene-edited mice with BRD3-linked fluorescent probes, we may be able to better address this issue. However, based on the current results, we speculate that the BRD3 expression in retinal microglial cells of EAU may not have significant changes. This observation is consistent with previous reports that BRD3 may not play a role exclusively based on its expression. For example, BRD3 protein has a disordered structure and can undergo liquid-liquid separation; receptor tyrosine kinase TYRO3 can catalyze BRD3 phosphorylation through cytoplasmic nuclear translocation; and BRD3 can influence the function of non-coding RNA.^22^^,^^30^ In addition, BRD3 may directly bind to chromatin remodeling-related proteins,^31^ suggesting that its functional role extends beyond its expression level and warrants further investigation. Future studies should focus on confirming the degradation specificity of PROTACs synthesized using different BRD3 inhibitors. From another perspective, developing PROTACs containing the other two BET protein degraders could be one of the future directions. Furthermore, although we observed the downregulation of BRD2 in the retinal results, we verified through rigorous *in vitro* experiments that BRD3, rather than BRD2, is the degradation target of D072. Since BRD2 has been previously reported to be associated with the initiation of inflammation, we speculate that degradation of BRD3 inhibits the retinal inflammatory environment, thereby indirectly affecting the expression level of BRD2.

After degrading BRD3 with D072 or interfering with its expression, we found that BRD3 actively participated in the inflammatory response. We focused on the downstream changes after BRD3 degradation as a recognition protein for acetylation. Additionally, D072-induced BRD3 degradation caused a series of histone acetylation modifications, especially H3K18ac, which was upregulated in LPS-stimulated microglia *in vitro* and EAU activated amebic microglia *in vivo*, but this upregulation could be inhibited by D072. We hypothesized that the degradation of BRD3 exposes the acetylation sites to the combined actions of acetyltransferase and deacetylases. Our findings were further supported by the observation that the HDAC inhibitor sodium butyrate could upregulate H3K18ac, suggesting a general mechanism by which BET family proteins influence histone acetylation modifications. Unlike BRD4, BRD3 has not been reported to have acetyltransferase activity,^32^ and the possibility that other types of deacetylases may play a more prominent regulatory role remains open for further study. In addition, acetyltransferases may also play a significant role in this process. Notably, CBP/p300, a class of extensively studied acetyltransferases, has been shown to exert critical regulatory functions in tumor metastasis and inflammatory responses through modulating cellular histone modification levels. Further investigation is warranted to elucidate the precise molecular mechanisms underlying their involvement in these pathological processes.

In this study, we reported that D072 has a certain anti-inflammatory effect on uveitis and LPS-stimulated microglia by degrading BRD3. This finding suggests that D072/BRD3 may be a potential target for the treatment of uveitis and other autoimmune inflammatory diseases. Nevertheless, the potential role of D072 in mammalian models has yet to be explored, and the precise mechanisms by which BRD3 influences uveitis pathogenesis remain incompletely understood. Further studies are needed to better elucidate BRD3’s role and to explore the applicability of D072 as a clinical therapeutic strategy for uveitis. In summary, we identified a novel PROTAC, D072, which showed anti-inflammatory effects *in vivo* by inhibiting microglia activation. D072 specifically degrades BRD3, leading to the downregulation of H3K18ac without affecting BRD2 or BRD4. This downregulation subsequently alters the expression of genes related to inflammation/metabolism, including iNOS, HK3, CCL5, and CXCL10. Further studies have shown that HDACs are involved in the regulation of H3K18ac, and CCL5 is the downstream target of this pathway. Taken together, our findings provide potential therapeutic targets and drug candidates for uveitis treatment.

### Limitations of the study

This study identified a BRD3 degrader that reduces the inflammatory level of microglia cells in uveitis by decreasing H3K18ac. This provides a potential approach for the treatment of uveitis; however, there are still some limitations that are worth discussing. First of all, the degradation effect of PROTAC is highly dependent on the species conservation of the protein. The BRD3-selective degradation effect of D072 should be re-verified in human cells through experiments. If necessary, the structure should be optimized. Second, the intravitreal injection of D072 lacks specific cell targeting and braking mechanisms. Although no significant toxic side effects were observed during our testing period, further investigation is needed to determine its long-term effects. Last but not least, the mechanism of BRD3 in microglia still requires further investigation. Although we observed the significant role of BRD3 degradation in stabilizing the expression level of H3K18ac, I also found that the BRD3 protein levels in the uveitis retina and LPS-stimulated microglia did not show any significant changes. This is the direction we will strive for in the future. We speculate that it may be related to the post-translational modification of BRD3 or the distribution of cells.

## Resource availability

### Lead contact

Further information and requests for resources and reagents should be directed to and will be fulfilled by the lead contact, Shengping Hou (sphou828@163.com).

### Materials availability

This study did not generate new unique reagents.

### Data and code availability

• This paper does not report original code, but it is available from the lead contact upon request.
• CUT&Tag of H3K18ac data have been deposited at China National Center for Bioinformation, GSA number (CRA031344) and project number (PRJCA048185) are publicly available as of the date of publication. Accession numbers are listed in the key resources table. Please cite https://ngdc.cncb.ac.cn/gsa/search?searchTerm=CRA031344.

## Acknowledgments

This work was supported by the National Natural Science Foundation Project of China (82271078, 82371045), Beijing Municipal Public Welfare Development and Reform Pilot Project for Medical Research Institutes (PWD&RPP-MRI, JYY2023-6), and Beijing Young Scholar Program (no. 076).

## Author contributions

Z.Z., Y.L., and S.H. conceived and designed research; Z.Z., N.S., R.L., W.L., and Q.Z. performed the experiments; Z.Z., N.S., T.L., H.Y., and P.Y. analyzed data; Z.Z., S.H., H.Y., and Y.L. wrote and revised the paper; S.H., P.Y., and Y.R. supervised the experiments and revised the manuscript; S.H. conceptualized the study and acquired funding.

## Declaration of interests

The authors declare no competing interests.

## STAR★Methods

### Key resources table

**Table:** 

REAGENT or RESOURCE	SOURCE	IDENTIFIER
**Antibodies**		

IBA1	Wako	019-19741; RRID: AB_839504
H3K18ac	Abcam	ab1191; RRID:AB_298692
iNOS	Santa Cruz	sc-7271; RRID:AB_2891105
COX-2	Santa Cruz	sc-19999; RRID:AB_627284
galectin-3	Santa Cruz	sc-374253; RRID:AB_10987671
BRD2	Santa Cruz	sc-514103; RRID:AB_3720459
BRD3	Santa Cruz	sc-81202; RRID:AB_1119692
BRD4	Abcam	ab128874; RRID:AB_11145462
BRD3	Abcam	ab50818; RRID:AB_868478
Histone H3	PTM BIO	PTM-6621; RRID:AB_3720450
Histone H4	PTM BIO	PTM-1015RM; RRID:AB_3101866
Acetyl-Histone H3 (Lys56)	PTM BIO	PTM-162; RRID:AB_3720454
Acetyl-Histone H3 (Lys27)	PTM BIO	PTM-116RM; RRID:AB_3712824
Acetyl-Histone H3 (Lys18)	PTM BIO	PTM-114RM; RRID:AB_3076698
Acetyl-Histone H3 (Lys14)	PTM BIO	PTM-157; RRID:AB_2722570
Acetyl-Histone H3 (Lys9)	PTM BIO	PTM-112RM; RRID:AB_3719481
Acetyl-Histone H4 (Lys16)	PTM BIO	PTM-122; RRID:AB_3170228
Acetyl-Histone H4 (Lys5)	PTM BIO	PTM-163; RRID:AB_3717383
HRP-conjugated AffiniPure goat anti-mouse IgG	Proteintech	SA00001-1; RRID:AB_2722565
HRP-conjugated AffiniPure goat anti-rabbit IgG	Proteintech	SA00001-2; RRID:AB_2722564

**Biologlcial sample**		

Bv2 cell line	Fuheng biology	Fh0355
HMC3 cell line	Fuheng biology	Fh1111

**Chemicals, peptides, and recombinant proteins**		

HMC3 complete culture medium	Fuheng biology	FH-HMC3
BV2 complete culture medium	Fuheng biology	FH-BV-2
human IRBP651–670 (LAQGAYRTAVDLESLASQLT)	Sangon Biological Engineering Technology &Services	N/A
complete Freund’s adjuvant	Sigma-Aldrich	N/A
M. tuberculosis strain H37Ra	BD Biosciences	N/A
DMSO	Sigma-Aldrich	V900090 MSDS
FAS eye fixation solution	Servicebio	#G1109
BrdU	Invitrogen	00-0103
borate buffer	Sigma-Aldrich	08059
lipopolysaccharide	Sigma-Aldrich	93572-42-0
TRIzol reagent	Invitrogen	15596026CN
Lyse buffer	Beyotime	P0013B
crystal violet	Beyotime	Y268091-100g
Sodium butanoate	Targetmol	T1393
EX-527	Targetmol	T6111
SIRT-IN-2	Targetmol	T12929
SIRT6-IN-5	Targetmol	T24793
97491	Targetmol	T39233

**Critical commercial assays**		

BCA Protein Assay Kit	NA	P0009
immunoprecipitation kit	Abcam	ab206996
cell counting kit-8 (CCK-8) assay	MedChemExpress	HY-K0301
ELISA kit	Elabscience	N/A
ECL kit	4A biotech	N/A

**Software and algorithms**		

GraphPad Prism	GraphPad Software	RRID: SCR_002798
FlowJo	BD	RRID: SCR_008520
ImageJ	NIH	RRID:SCR_003070

**Deposited data**		

CUT&Tag of H3K18ac	this paper	CRA031344

### Experimental model and study participant details

#### Animals

Female C57BL/6J mice (6–8 weeks old) were provided by The Experimental Animal Center of Chongqing Medical University and were kept in an environment that was pathogen-free. Approval of the experiments was given by the Ethics Committee of the First Affiliated Hospital of Chongqing Medical University (No. 2019-296). The ARVO Statement for the Use of Animals in Ophthalmic and Vision Research was followed by all procedures.

#### Experimental models sections

##### Biological samples section

The human microglial cell line HMC3 was purchased from Fuheng Biology (FH1111) and maintained in our laboratory with HMC3 complete culture medium (FH-HMC3), which contains Eagle’s minimum essential medium, 10% fetal bovine serum, and 1% penicillin/streptomycin. The mice microglial cell line BV2 (FH0355) was purchased from the Fu Heng Biology and maintained in our laboratory with BV2 complete culture medium (FH-Bv-2) containing DuIbecco’s modified eagIe’s medium with 10% fetal bovine serum and 1% penicillin/streptomycin. All these cells were cultured at 37 °C with 5% CO_2_.

### Method details

#### EAU induction and D072 intravitreal injection

Female C57BL/6J mice were subcutaneously immunized with 500 μg of human IRBP651–670 (LAQGAYRTAVDLESLASQLT) (Sangon Biological Engineering Technology &Services Co., Ltd., Shanghai, China) dissolved in PBS containing 20% DMSO (V900090 MSDS, Sigma-Aldrich, USA) and emulsified with an equal volume of complete Freund’s adjuvant (Sigma-Aldrich, St. Louis, MO, USA) containing M. tuberculosis strain H37Ra (BD Biosciences, New Jersey, USA). Subsequently, the mice were injected intraperitoneally with 1 μg of *Bordetella pertussis* toxin (Sigma-Aldrich, St. Louis, Missouri, USA). The EAU animals with clinical symptoms confirmed by a slit lamp were selected. On the 9^th^ day after EAU induction, mice were administered a single intravitreal injection of D072 (300 μM). D072 was dissolved in DMSO and diluted with PBS. Mice injected with DMSO diluted proportionally in PBS serve as controls.

#### PROTAC library synthesis

The synthesis of PROTAC library involved the selection of small molecule inhibitors targeting distinct biological markers, linkers, and E3 ubiquitin ligase ligands. By utilizing advanced combinatorial chemistry techniques to conjugate these components, we successfully synthesized an innovative and diverse PROTAC library. Detailed procedures for the chemical synthesis of selected PROTAC D072 are provided in Figure S1. Please refer to procedures for the chemical synthesis of PROTAC D072. for the detailed information.

#### Procedures for the chemical synthesis of PROTAC D072

(1) 88 mg of raw material 1 was added to a 10 mL round-bottom flask and dissolved in 2 mL of N, N-dimethylformamide. Subsequently, 88 mg of 3-(prop-2-yn-1-yloxy) phenol and 275 mg of potassium carbonate were sequentially added. The reaction mixture was maintained at 110°C with stirring for approximately 2 h, until thin-layer chromatography (TLC) indicated the disappearance of raw material 1. The reaction mixture was then cooled down to room temperature. The reaction mixture was added to water followed by several times of extraction with ethyl acetate, the organic phases were combined and dried over anhydrous sodium sulfate. After evaporating the solvent, the residue was dissolved in 10 mL of a methanol/water (3:1) mixture along with 100 mg of activated iron powder and 50 mg of ammonium chloride. The mixture was refluxed at 80°C for 1.5 h with continuous stirring. After cooling to room temperature, it was filtered through diatomaceous earth to remove the remaining iron powder. Water was added to the filtrate, and it was extracted three times with ethyl acetate (20 mL each). The organic phases were combined, dried with anhydrous sodium sulfate, and the solvent was removed by rotary evaporation. The residue was purified using a 200–300 mesh silica gel column with dichloromethane (20:1) as the mobile phase, yielding 56 mg of compound 4 with a 73% yield. 50 mg of compound 4 was added into a 10 mL round-bottom flask and dissolved in 10 mL of dichloromethane, which was then placed in an ice-water bath. 330 mg of ethyl sulfonyl chloride and 360 μL of triethylamine were sequentially added, and the mixture was stirred at low temperature until the raw material had completely disappeared. The reaction mixture was then added to 150 mL of water and extracted three times with dichloromethane (20 mL each). The organic phases were combined, dried with anhydrous sodium sulfate, and the solvent was removed by rotary evaporation. The residue was transferred to a 10 mL round-bottom flask, to which 22 mg of potassium fluoride, 90 mg of potassium carbonate, and 43 mg of 1,3-dihydroxybenzene were added sequentially. The mixture was stirred at 110°C for 2 h. After cooling to room temperature, the mixture was added to 150 mL of water and extracted three times with ethyl acetate (20 mL each). The organic phases were combined, dried with anhydrous sodium sulfate, and the solvent was removed by rotary evaporation. The residue was purified using a 200–300 mesh silica gel column with dichloromethane (30:1) as the mobile phase, yielding 45 mg of compound 5 with a 73% yield. ^**1**^**H-NMR (400 MHz, CDCl3) δ (ppm)** 10.66 (s, 1H), 7.50 (s, 1H), 7.29 (m, 1H), 7.15-7.05 (m, 3H), 6.60 (d, J = 8.60 Hz, 1H), 6.48-6.41 (m, 3H), 4.57 (s, 2H), 3.56 (s, 3H), 3.19 (q, J = 6.96 Hz, 2H), 1.42 (t, J = 7.20 Hz, 3H);
(2) 2-(2,6-dioxopiperidine-3-yl)-4-fluorophenylfuroxan-1,3-dione (Compound 6), 2.0 equivalents of DIEA, an appropriate amount of N, N-dimethylformamide as the solvent, and 2.0 equivalents of amine starting material with either an azido or carboxylic acid end group were added to a round-bottom flask. The reaction mixture was stirred at 80°C for 3 h and was quenched by adding saturated brine. The mixture was extracted with ethyl acetate for 3 to 5 times. The organic phases were combined, dried with anhydrous sodium sulfate, and the solvent was removed by rotary evaporation. The residue was purified using a 200–300 mesh silica gel column with a mobile phase of ethyl acetate: petroleum ether at a ratio from 1:1 to 3:1, achieving a yield of 25%.
(3) 15 mg of compound 5 and 15 mg of ligand compound 7 were placed in a 5 mL round-bottom flask, followed by the addition of 50 mg of sodium ascorbate and 2 mL of N,N-dimethylformamide/water (5:1) mixture. Finally, 2 mg of anhydrous copper sulfate was added, and the mixture was reacted at room temperature for 6 h. The reaction was quenched with saturated brine, followed by the extraction with ethyl acetate for 3 to 5 times. The organic phases were combined and dried with anhydrous sodium sulfate, and the solvent was removed by rotary evaporation. The residue was purified using a 200–300 mesh silica gel column with a mobile phase of dichloromethane ranging from 30:1 to 10:1, yielding 14 mg of the target compound with a 47% yield. ^**1**^**H-NMR (400 MHz, CDCl3) δ (ppm)** 10.28 (s, 1H), 9.25 (s, 1H), 7.54 (s, 1H), 7.49-7.45 (m, 2H), 7.27 (s, 1H), 7.25-7.22 (m, 2H), 7.13-7.04 (m, 4H), 6.87 (d, J = 8.52 Hz, 1H), 6.63-6.60 (m, 1H), 6.46-6.44 (m, 2H), 6.40-6.39 (m, 1H), 6.21 (m, 1H), 5.06 (s, 2H), 4.93-4.90 (m, 1H), 4.33 (t, J = 7.20 Hz, 1H), 3.58 (s, 3H), 3.27-3.16 (m, 4H), 2.86-2.76 (m, 3H), 2.12-2.11 (m, 1H), 1.89-1.86 (m, 2H), 1.66-1.63 (m, 2H), 1.42 (t, J = 7.36 Hz, 3H), 1.32-1.25 (m, 14H); LC-MS (ESI^+^): m/z calculated for C49H56N9O9S (M + H)^+^: 946.38 found 947.35.

Related to Figure S1.

#### D072 intravitreal injection

On the 9^th^ day after EAU induction, mice were administered a single intravitreal injection of D072 (300 μM). D072 was dissolved in DMSO and diluted with PBS. Mice injected with DMSO diluted proportionally in PBS serve as controls.

#### Hematoxylin and eosin (H&E) staining

On the 14^th^ day after EAU induction, sacrifice the mouse and obtain eye balls, fixed in FAS eye fixation solution (Servicebio#G1109) for 24 h. Ethanol gradient dehydration, paraffin embedding. Slices of 4-8UM were cut parallel to the optic nerve for HE staining.

#### Slit lamp, fundoscopy, and histopathological assessment

The clinical symptoms of EAU were assessed using a slit lamp examination on the 14^th^ day following models. Three independent observers blindly assessed the clinical severity of ocular inflammation and scored half-point increments on a scale of 0–5 based on five independent criteria and Caspi’s criteria.^33^ Fundus photos were taken with an ophthalmoscope and evaluation of grades 0–4, following Caspi’s criteria.^34^ Hematoxylin and eosin (H&E) were used to stain the Paraffin section for histopathological evaluation of grades 0–4, following Caspi’s criteria.^33^

#### Immunofluorescence

For retinal flat mounts, the eyeballs were immersed in 4% paraformaldehyde for 6 h, cut into flat mounts, blocked with 3% goat serum and 0.3% Triton X-100 for 1 h at room temperature, and then incubated with primary antibodies at 4°C overnight. Next, the retinas were washed three times with PBS and incubated with Alexa Fluor dyes conjugated secondary antibodies. Images were taken by confocal microscopy (Zeiss, Germany).

Primary antibodies included IBA1 (1:1000), H3K18ac (1:500), IBA1 (1:1000), and DAPI. Secondary antibodies conjugated to Alexa Fluor 488, Alex a Fluor 568 and Alexa Fluor 647 (Molecular Probes) were 1:300 diluted.

#### Cell treatment

BV2 cells were treated with freshly prepared serum-free medium containing given concentrations of D072 and stimulated with or without lipopolysaccharide (LPS; 1 μg/mL) for 24 h after 12 h serum starvation.

#### Cell transfection

Small interfering RNA (siRNA) targeting BRD3 (Thermo Fisher Scientific) was used for BRD3 knockdown in BV2 cells. The sequences of siRNAs are shown in key resources table. A BRD3 overexpression lentivirus containing the BRD3 coding sequence (CDS) was used to induce BRD3 overexpression in BV2 cells.

#### BrdU incorporation assay

A single dose of BrdU (50 mg/kg, Invitrogen, 00–0103) was administered to the animals 24 h prior to sacrifice. Whole-mount retinas were denatured by 2 M hydrochloric acid for 20 min at RT, followed by renaturation with 0.01 M borate buffer (pH 8.2, Sigma-Aldrich 08059) at RT for 30 min before 0.01 M PBS rinse for 10 min. The immunohistochemistry was then carried out afterward.

##### Quantitative real-time PCR

Total RNA was extracted from EAU tissues and BV2 cells using the TRIzol reagent (Invitrogen, San Diego, California, USA), as per the instructions. RT Master Mix for qPCR (MedChemExpress, USA) and SYBR Green qPCR Master Mix (MedChemExpress, USA) were utilized to measure the levels of mRNA. All results were normalized with β-actin. The primer sequences used for the targeted genes are shown in key resources table.

##### Western blotting analysis

Lyse buffer (Beyotime, Shanghai, China) was utilized to extract proteins from cells and retinal tissues, and then concentration was determined using a BCA Protein Assay Kit (Beyotime). Equivalent amounts of protein samples were separated by 4–20% (wt/vol) SDS-PAGE (ACE, Changzhou, China) and transferred to PVDF membranes (Millipore, Billerica, MA, USA). The membranes were blocked with 4% skim milk for 1.5 h at room temperature and then incubated with primary antibodies overnight at 4°C. The membranes were washed once in TBST and incubated with secondary antibodies for 1 h at room temperature. The band was visualized using an ECL kit (4A biotech, Beijing, China), and band densitometry was quantified using ImageJ software. The primary antibodies used in this study were as follows: iNOS (1:800), COX-2 (1:500), galectin-3 (1:500), BRD2 (1:500), BRD3 (1:800), BRD4 (1:1000), BRD3 (1:50), Histone H3 (1:1000), Histone H4 (1:1000), Acetyl-Histone H3 (Lys56) (1:1000), Acetyl-Histone H3 (Lys27) (1:1000), Acetyl-Histone H3 (Lys18) (1:1000), Acetyl-Histone H3 (Lys14) (1:1000), Acetyl-Histone H3 (Lys9) (1:1000), Acetyl-Histone H4 (Lys16) (1:1000), Acetyl-Histone H4 (Lys5) (1:1000). The secondary antibodies used included HRP-conjugated AffiniPure goat anti-mouse IgG (1:6000) and HRP-conjugated AffiniPure goat anti-rabbit IgG (1:6000).

##### Immunoprecipitation

The immunoprecipitation of the corresponding proteins was performed by the immunoprecipitation kit (Abcam, ab206996). Firstly, the protein of BV2 cells was isolated by the non-denaturing lysis buffer containing protease inhibitors. Then, proteins were incubated with anti-BRD3 antibody (ab50818, Abcam, UK) or IgG isotype control (Beyotime, China) overnight at 4°C, and the protein complexes were captured by protein A/G Sepharose beads (ab206996, Abcam, UK) and eluted with SDS-PAGE loading buffer. Finally, western blot analysis was performed to determine eluted proteins.

##### Cell proliferation assays

Cell proliferation was evaluated using cell counting kit-8 (CCK-8) assay (HY-K0301, MedChemExpress, USA) following the manufacturer’s instructions. Firstly, 8000 cells were seeded in 96-well plates and cultured in a CO_2_ incubator at 37°C for 24 h, followed by the addition of different compounds. Then, CCK-8 solution was added at the indicated times (0, 24h), and the plate was incubated at 37°C for 2 h. Lastly, the absorbance was measured at 450 nm.

##### Enzyme-linked immunosorbent assay (ELISA)

The cell supernatant was harvested, centrifuged, and then stored at −80°C. The CCL-5 concentration was measured using ELISA kits (Elabscience, Wuhan, China).

##### Transwell assays

The system used is a 24-well transwell system (Corning, NY, USA), which has chambers with 8 μm pores. The upper compartment was seeded with 1x10^5^ BV2 cells from various groups, and 10% FBS medium was added to the lower chamber. The lower chamber was filled with or without LPS (1 μg/mL, Sigma) and D072 (10 nM) after 24 h of incubation at 37°C. After 24 h, PBS was used to wash the BV2 cells three times, followed by 4% paraformaldehyde fixation for 20 min, and staining with crystal violet (Beyotime, China). After removing the non-migrating cells on the upper side of the membrane, we took images of the stained cells on the lower side and used ImageJ to calculate them.

##### CUT & Tag analysis

In accordance with the manufacturer’s instructions, the CUT&Tag assay was performed using a Hyperactive Universal CUT&Tag Assay Kit from Illumina (Vazyme, TD903-01). Briefly, BV2 cells were randomly distributed into three groups: the control group cultured in serum-free medium, and the experimental group treated with LPS+DMSO and the treated group incubated LPS with D072 for 24 h. Collected cells were bound to Concanavalin A-coated magnetic beads, permeabilized using Digitonin, and incubated with H3K18ac antibodies (abcam, ab178945). Next, pA-Tn5 transposase was added. DNA was extracted, amplified, and purified using transposon and tagmentation to construct a library. An Illumina NovaSeq 150 PE platform was used for data analysis.

### Quantification and statistical analysis

IBM SPSS Statistics 20.0 (Chicago, USA) was used to analyze all the data, which were expressed as mean ± SD. The construction of all the figures was done with GraphPad Prism version 9.0 software (San Diego, USA). For data conforming to normality and homogeneity of variance, unpaired Student’s *t* test was employed to compare two sets of samples, and one-way ANOVA followed by Bonferroni’s test was used in multiple groups. Otherwise, a nonparametric test was applied via the Mann-Whitney U test. The statistical significance of differences between groups was determined when the *p* value was <0.05. (*∗p < 0.05, ∗∗p < 0.01, ∗∗∗p < 0.001)*. n indicates the number of experimental repetitions. The specific statistics and details can be found in each figure legends.
