# Supplementary material for: BRD3 PROTAC degrader targets H3K18ac to alleviate retinal microglia-driven uveitis
Source: iScience. 2025 Dec 22;29(2):114526. doi: 10.1016/j.isci.2025.114526 (PMC12828559; doi:10.1016/j.isci.2025.114526)
Supplement: Document S1. Figures S1–S4 [file mmc1.pdf]

iScience, Volume *29*

## Supplemental information

# BRD3 PROTAC degrader targets H3K18ac

# to alleviate retinal microglia-driven uveitis

**Zhi Zhang, Tianlong Lan, Yongbo Liu, Hui Yang, Nan Shu, Ruonan Li, Wanqian Li, Qian Zhou, Peizeng Yang, Yu Rao, and Shengping Hou**

**Supplemental information**

**Document S1. Figures S1-S4**

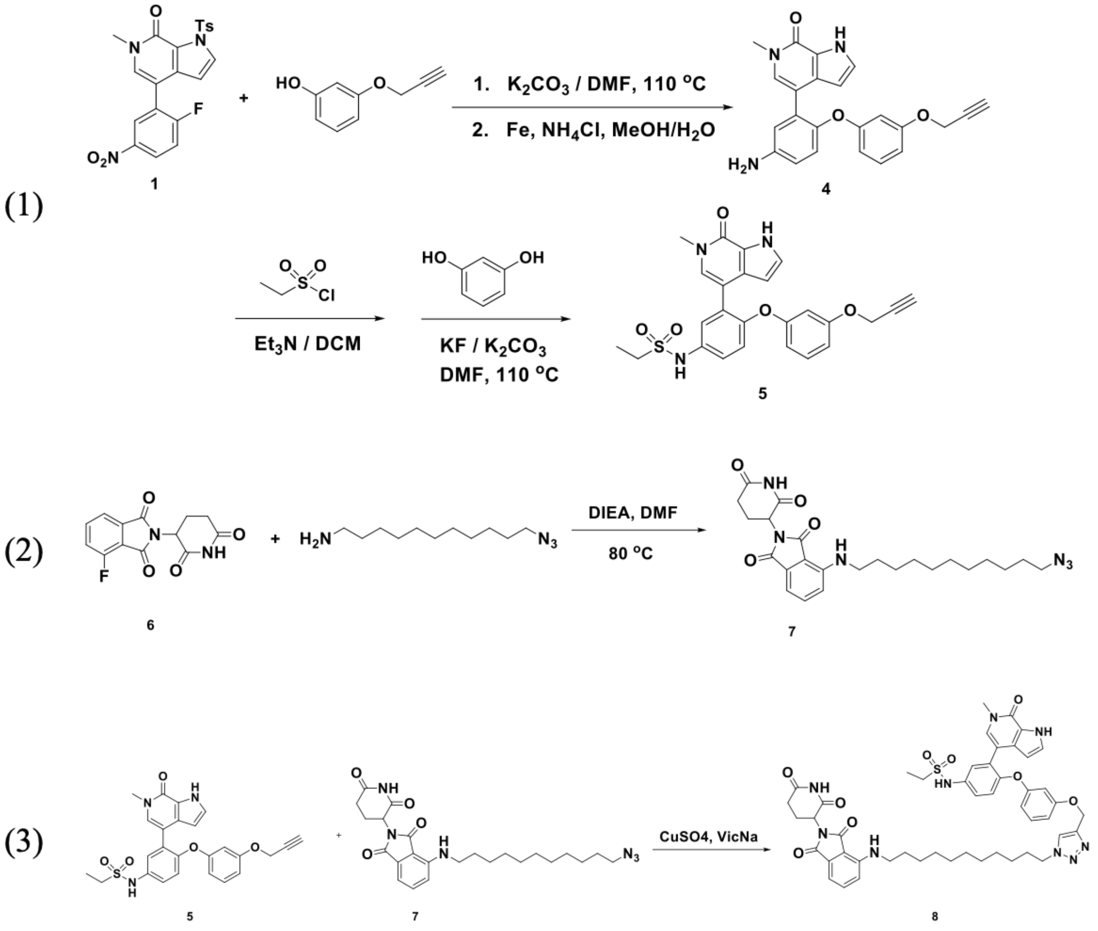


***Figure S1*. The chemical synthesis of D072.** (1) Abbv075 is obtained by a four-step synthesis (See Methods for details); (2) Pomalidomide is ligated with an alkyl chain-based PROTAC linker; (3) PROTAC D072 was generated by an azide-alkyne click chemistry of compound 5 and compound 7.

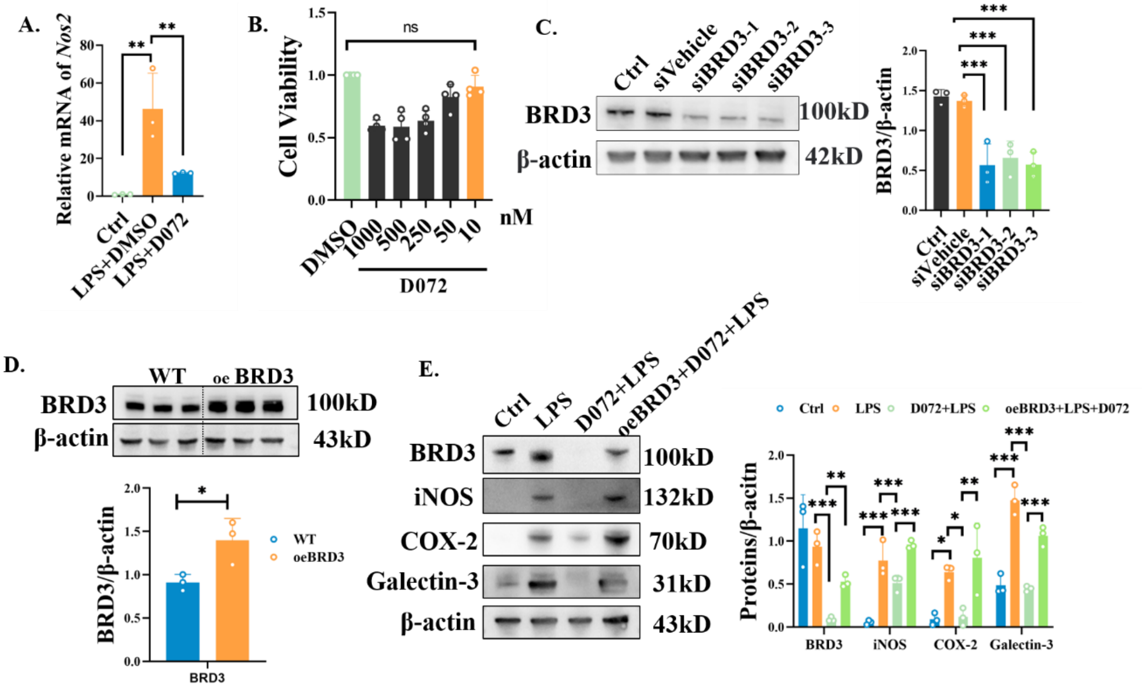


F.

| ID | D043 | D049 | D034 | D032 | D019 | D014 |
|---|---|---|---|---|---|---|
| Cell Viability | 21% | 21% | 27% | 30% | 31% | 32% |
| **ID** | **D011** | **D047** | **D009** | **D072** | **D013** | **D008** |
| Cell Viability | 32% | 33% | 35% | 35% | 38% | 39% |

***Figure S2. In vitro* experiments of drug screening and BRD3 intervention. (A)** Quantification of the mRNA levels of iNOS after D072 treated for 6h in LPS-stimulated BV2 cells (n=3/group; mean ± SD; ***P<0.01*; one-way ANOVA); **(B)** Cell viability analysis of BV2 cells under the treatment of different concentrations of D072 by CCK8 assay (n=4/group; mean ± SD; ns *P>0.05*; one-way ANOVA); **(C)** Left: Representative western blot images of BRD3 after siRNAs or vehicle treatment in BV2 cells. Right: Quantification of the relative fold changes of the left (n=3/group; mean ± SD; ****P<0.001*; one-way ANOVA); **(D)** Left: Representative western blot images of BRD3 after overexpression BRD3 or vehicle treatment in BV2 cells. Right: Quantification of the relative fold changes of the left (n=3/group; mean ± SD; **P<0.05*; independent-samples T test); **(E)** Western blotting analysis of the protein levels of iNOS, COX-2, Galectin-3, and BRD3 in different groups. Left: Representative western blot images of iNOS, COX-2, Galectin-3, and BRD3 in different groups. Right: Quantification of the relative fold changes of the left (n=3/group; mean ± SD; **P<0.05*, ***P<0.01*, ****P<0.001*; one-way ANOVA). **(F)** The cell proliferative activity of the top 12 selected compounds was evaluated by CCK-8 assay (n=1). HMC3 cells were incubated with each compound for 48 hours. The cell proliferation inhibition of each compound is expressed as the number of treated cells relative to the untreated control.

Fig S3

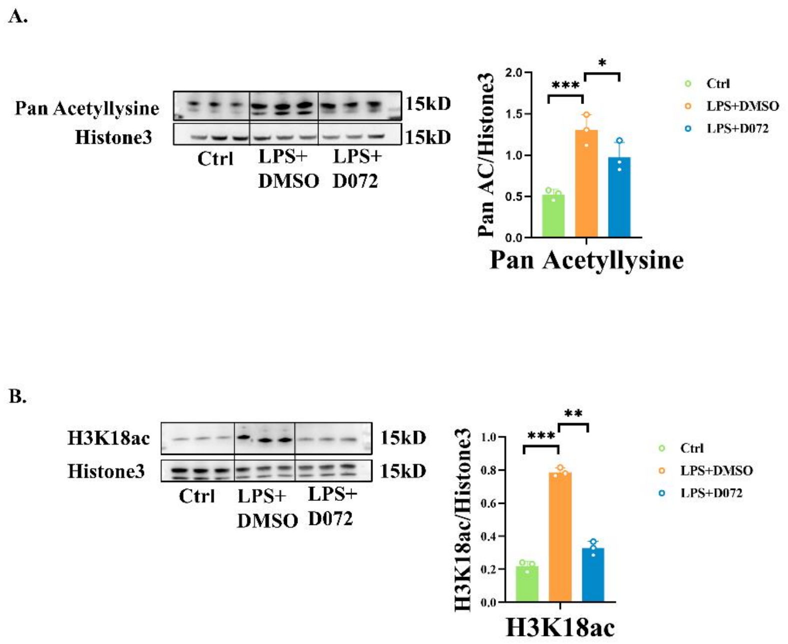


***Figure S3.*** **The changes in histone acetylation modifications of BV2 cells under LPS stimulation and D072 treatment (A, B)** Left: Representative western blot images of Pan Acetyllysine (B) and H3K18ac (C) between control, LPS+DMSO, and LPS+D072 treatment in BV2 cells. Right: Quantification of the relative fold changes of the left (n=3/group; mean ± SD; ****P<0.001*; one-way ANOVA).

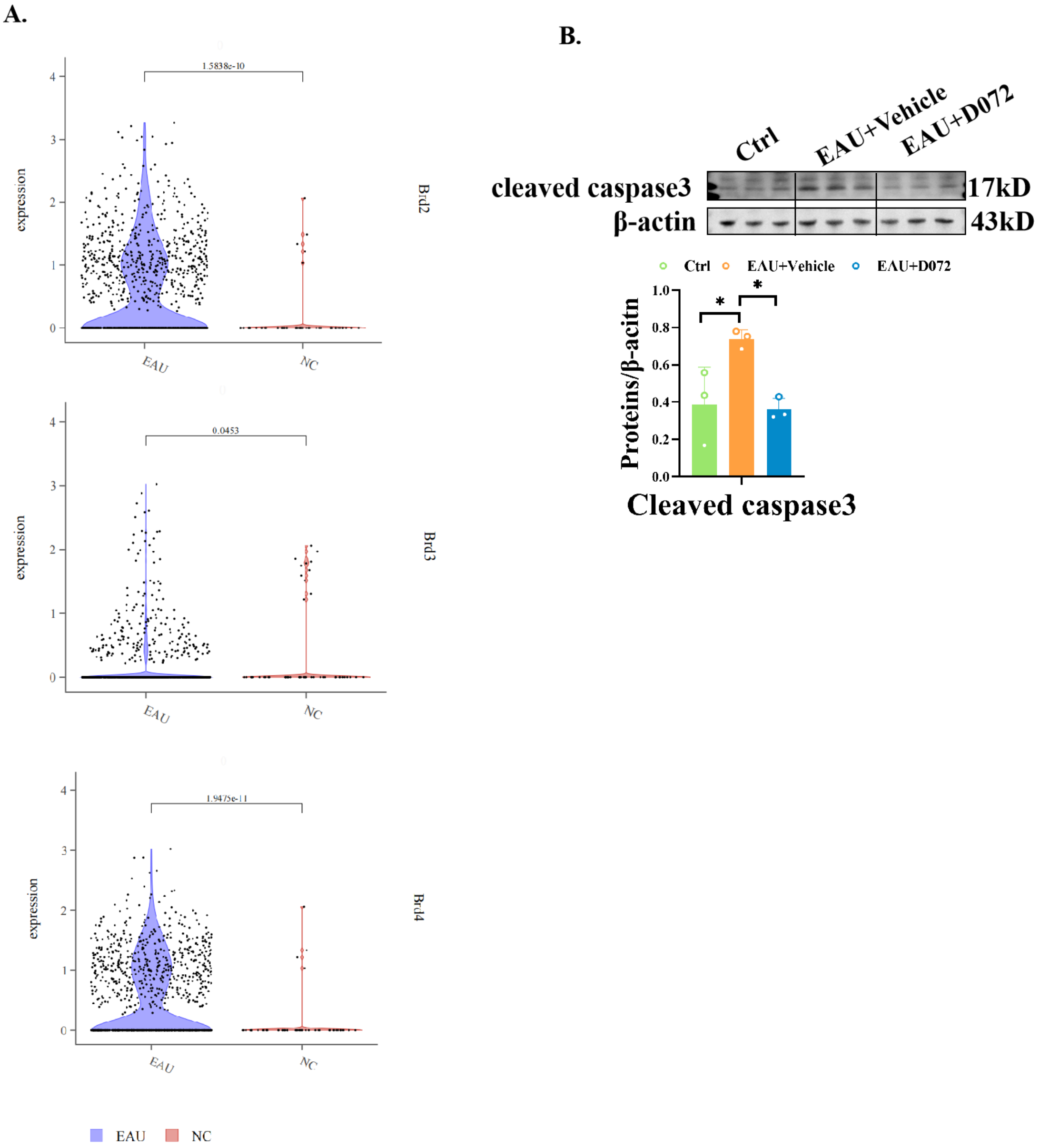


***Figure S4.*** **BRD2, BRD3 and BRD4 in the single-cell data results of retinal microglia in EAU and the retinal toxicity experiment of D072. (A)** Single-cell data of mRNA levels of BRD2, BRD3, and BRD4 in retinal microglia between EAU and control groups (independent-samples T test)**. (B)** Upper: Representative western blot images of cleaved caspase 3 between control, EAU+Vehicle, and EAU+D072 treatment groups. Lower: Corresponding quantification of the relative fold changes (n=3/group; mean ± SD; **P<0.05*; one-way ANOVA).
